# Angiographic Diagnosis of a Meckel’s Diverticulum in a 26-month-old Boy

**DOI:** 10.1097/PG9.0000000000000143

**Published:** 2021-12-03

**Authors:** Amit Patel, Michael R. Accord, Peter Mattei, Tricia R. Bhatti, Christopher M. Sande, Lindsey Albenberg

**Affiliations:** From the * Division of Gastroenterology, Hepatology, and Nutrition, Children’s Hospital of Philadelphia, Philadelphia, Pennsylvania; † Rowan University School of Osteopathic Medicine, Stratford, New Jersey; ‡ Department of Radiology; § Department of General, Thoracic, and Fetal Surgery; ∥ Department of Pathology and Laboratory Medicine, Children’s Hospital of Philadelphia, Philadelphia, Pennsylvania

A 26-month-old boy presented with recurrent hematochezia. Abdominal examination was normal. Laboratory findings showed hemoglobin of 7.5 g/dL. Abdominal ultrasound (US) for intussusception revealed a short (0.3 cm) segment of wall thickening in the distal ileum and Meckel’s scan was negative. The patient underwent two additional Meckel’s scans, tagged red blood cell scan, CT-angiography, upper endoscopy and colonoscopy, and video capsule endoscopy, but had normal results. Superior mesenteric angiography was performed revealing an area of abnormal vasculature in the terminal ileum (Fig. 1). Subsequently, exploratory laparotomy showed an obvious Meckel’s diverticulum (MD), and Meckel’s diverticulectomy was performed (Fig. 2).

**FIGURE 1.**
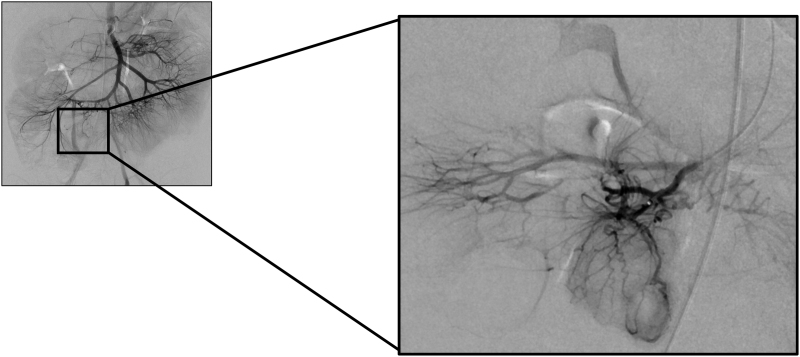
Meckel's Diverticulectomy.

**FIGURE 2.**
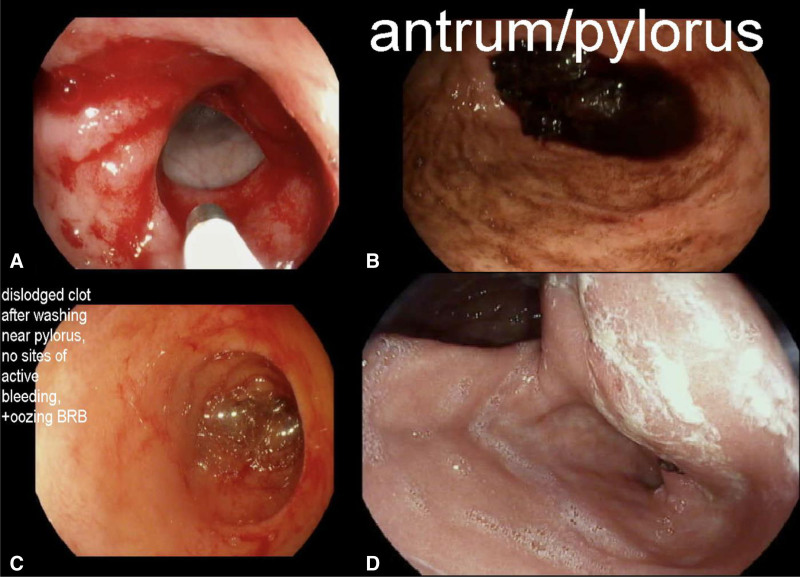
Meckel’s Diverticulectomy. A) Gross specimen of MD removed from patient and (B) histological examination of MD with the presence of ectopic gastric mucosa.

MD is the most common congenital malformation of the gastrointestinal tract, occurring in approximately 2% of the population (1). The gold standard in diagnosing a MD is a Meckel’s scan which detects the accumulation of a radioisotope, Technetium-99m. However, false negatives do occur and this is likely due to insufficient gastric mucosa in the MD (2). The literature on typical angiographic findings for diagnosis of MD are limited to a few individual cases (3–6). Our patient exhibited multiple abnormal corkscrew-like arteries of the ileocolic artery. Angiography can detect MD in the absence of acute bleeding through visualization of the vitelline artery (7).

## ACKNOWLEDGMENTS

A.P. was involved in conception and design, acquisition of data, drafting the work, final approval of the version to be published. M.R.A., P.M., T.R.B., and C.M.S. were involved in conception and design, critical revision of work, final approval of version to be published. L.A. was involved in conception and design, acquisition of data, critical revision of work, final approval of version to be published.
